# Diagnostic performance of AI-based models versus physicians among patients with hepatocellular carcinoma: a systematic review and meta-analysis

**DOI:** 10.3389/frai.2024.1398205

**Published:** 2024-08-19

**Authors:** Feras Al-Obeidat, Wael Hafez, Muneir Gador, Nesma Ahmed, Marwa Muhammed Abdeljawad, Antesh Yadav, Asrar Rashed

**Affiliations:** ^1^College of Technological Innovation, Zayed University, Abu Dubai, United Arab Emirates; ^2^NMC Royal Hospital, Khalifa City, United Arab Emirates; ^3^Internal Medicine Department, Medical Research and Clinical Studies Institute, The National Research Centre, Cairo, Egypt; ^4^EMS, Dubai, United Arab Emirates; ^5^Department of Computer Science, Edinburgh Napier University, Merchiston Campus, Edinburgh, United Kingdom

**Keywords:** artificial intelligence, hepatocellular carcinoma, HCC, diagnostic performance, AI models

## Abstract

**Background:**

Hepatocellular carcinoma (HCC) is a common primary liver cancer that requires early diagnosis due to its poor prognosis. Recent advances in artificial intelligence (AI) have facilitated hepatocellular carcinoma detection using multiple AI models; however, their performance is still uncertain.

**Aim:**

This meta-analysis aimed to compare the diagnostic performance of different AI models with that of clinicians in the detection of hepatocellular carcinoma.

**Methods:**

We searched the PubMed, Scopus, Cochrane Library, and Web of Science databases for eligible studies. The R package was used to synthesize the results. The outcomes of various studies were aggregated using fixed-effect and random-effects models. Statistical heterogeneity was evaluated using I-squared (I^2^) and chi-square statistics.

**Results:**

We included seven studies in our meta-analysis;. Both physicians and AI-based models scored an average sensitivity of 93%. Great variation in sensitivity, accuracy, and specificity was observed depending on the model and diagnostic technique used. The region-based convolutional neural network (RCNN) model showed high sensitivity (96%). Physicians had the highest specificity in diagnosing hepatocellular carcinoma(100%); furthermore, models-based convolutional neural networks achieved high sensitivity. Models based on AI-assisted Contrast-enhanced ultrasound (CEUS) showed poor accuracy (69.9%) compared to physicians and other models. The leave-one-out sensitivity revealed high heterogeneity among studies, which represented true differences among the studies.

**Conclusion:**

Models based on Faster R-CNN excel in image classification and data extraction, while both CNN-based models and models combining contrast-enhanced ultrasound (CEUS) with artificial intelligence (AI) had good sensitivity. Although AI models outperform physicians in diagnosing HCC, they should be utilized as supportive tools to help make more accurate and timely decisions.

## Introduction

Primary liver cancer is a challenging disease that was the second most common cause of cancer mortality globally in 2018, and it is the 7th most common type of cancer (Bray et al., 2018). Between 2020 and 2040, the number of new cases of liver cancer are predicted to increase by 55% per year (Rumgay et al., 2022). Liver cancer cells are differentiated into primary and secondary liver cancers according to the origin of the cancer cells. In primary liver cancer, the cancer originates within the liver itself, while secondary liver cancer is a result of the metastasis of other organs.

Primary liver cancer types include hepatocellular carcinoma (HCC), intrahepatic cholangiocarcinoma, hepatoblastoma, fibrolamellar carcinoma, angiosarcoma, and hemangiosarcoma (Astrologo et al., 2023). HCC accounts for approximately 75% of all liver cancer cases. Infection with hepatitis B and C is the major risk factor for HCC; however, other factors may play a role, such as aflatoxin exposure, alcohol consumption, and smoking (Chuang et al., 2009; Mittal and El-Serag, 2013). The treatment of hepatocellular carcinoma depends on several factors, including tumor size, cancer stage, extrahepatic metastasis, and the extent of vascular invasion. In general, patients with HCC have a poor prognosis, which is determined by the stage of liver disease, disease severity, and diagnosis timing. Therefore, early diagnosis is crucial for a better prognosis (Galle et al., 2018; Marrero et al., 2018).

Serum biomarkers, such as alpha-fetoprotein (AFP-L3), des-gamma-carboxy prothrombin (DCP), Golgi protein 73 (GP73), and glypican-3 (GPC3), have beneficial value for early diagnosis. Several trace chemicals, such as circulating tumor noncoding RNA (ct-ncRNA), cell-free DNA (cfDNA), circulating tumor DNA (ctDNA), and circulating tumor cells (CTCs), are released into biological fluids and could serve as valuable diagnostic agents (Di Tommaso et al., 2009; Toyoda et al., 2011; Guo et al., 2018; Choi et al., 2019). Fine-needle aspiration (FNA) biopsy is considered an additional confirmation test (Galle et al., 2018). Computed tomography (CT) and magnetic resonance imaging (MRI) are widely used in cancer monitoring and diagnosis. Nevertheless, their sensitivity and specificity for early HCC detection are relatively low, so liver-specific contrast agents are used to improve imaging accuracy. The combination of gadolinium-ethoxybenzyl diethylenetriamine pentaacetic acid (Gd-EOB-DTPA) and MRI improves the diagnosis of liver lesions, but this combination is not optimal for small lesion detection (Yu et al., 2014; Marrero et al., 2018). Furthermore, imaging techniques depend on observer interpretation, which represents a major source of error and misdiagnosis; therefore, artificial intelligence (AI)-based models have been developed to overcome this issue.

Radiomics analysis is a novel tool developed for extracting data from medical images and combined with imaging techniques for better performance (Lambin et al., 2012; Wang et al., 2023). Other AI models are based on machine learning, deep learning (DL), and convolutional neural networks (CNNs). The algorithms generated by machine learning must first undergo training on datasets to make predictions. Deep learning is a subset of machine learning that learns and extracts difficult data using multiple layers. Another technology is convolutional neural networks (CNNs), which are considered the ideal model for diagnosis because they can process complex visual data through multiple layers and filters. Different models have been developed in conjunction with traditional methods to optimize the diagnosis process. AI tools are unbiased, smart, cost-effective, and noninvasive, and their efficacy is comparable to that of humans (Yamashita et al., 2018; Saba et al., 2019; Awal et al., 2023). In this systematic review and meta-analysis, we aimed to evaluate the diagnostic performance of different AI models for the diagnosis of hepatocellular carcinoma in comparison with human expertise.

## Methods

### Literature search

This systematic review and meta-analysis was registered in PROSPERO; **CRD42024517634**; https://www.crd.york.ac.uk/PROSPERO/#recordDetails and conducted in compliance with the PRISMA (Preferred Reporting Items for Systematic Reviews and Meta-Analyses) statement (Moher, 2009). We searched electronic databases, including PubMed, Scopus, Cochrane, and Web of Science, through the 15th of February 2024. We used the relevant keywords and MeSH terms for artificial intelligence, machine learning, liver cancer, hepatocellular carcinoma, and diagnosis.

### Inclusion and exclusion criteria

Studies were included if they (1) compared the performance of the AI model with that of physicians and (2) reported the sensitivity of the model in diagnosing HCC. Studies were excluded if they (1) were non-English, (2) did not compare AI models with clinicians, (3) did not report the model’s ability to differentiate between HCC and other types of liver cancer, or (4) did not report the outcomes of interest.

We excluded reviews, correspondences, editorials, errata, case reports, animal studies, and conference abstracts. No restrictions were applied to the publication year.

### Study selection and data extraction

Two independent authors filtered the studies according to their titles and abstracts. The screening was assisted by Rayyan, an online software tool (Ouzzani et al., 2016). Disagreements were settled through discussions. The data were extracted by two independent authors using a standard data extraction sheet, and disagreements were resolved through discussion. We extracted the general characteristics of the included studies, such as the first author name, year of publication, country, sample size, aim of the study, model used, diagnostic technique used, and limitations of the study.

### Quality assessment

We used the QUADAS-AI tool to assess bias in the included studies. Two independent authors assessed the quality of the included results, and any discrepancies were resolved through discussion (Sounderajah et al., 2021). This tool addresses four main domains:

1. Subject selection domain: signaling questions evaluate the quality of input data, patient eligibility criteria, source of datasets, image preprocessing, and information about the scanner model.
2. The external validation process is evaluated in the index test domain.
3. Reference standard domain: this domain assesses the ability of the reference standard to classify the target condition correctly.
4. Flow and timing domain: evaluate whether the time between index testing and reference standardization is reasonable.

A study is considered to be at low risk of bias if all signaling questions are answered with “yes,” questions answered with “no” flag potential bias, and further discussion is required to reach a final decision. If sufficient data were not available, questions were answered with “unclear.”

### Statistical analysis

We used R version 4.2.2 (2022-10-31) and RStudio [version 2022.07.2 (2009–2022)] from RStudio, Inc. (R Core Team, 2022; RStudio Team, 2022). We conducted a meta-analysis of the sensitivity, specificity, and accuracy of AI models for the diagnosis of hepatocellular carcinoma (HCC) with the “metafor” package. The outcomes of the various studies were aggregated using fixed effects and random effects models because of the significant heterogeneity observed in the preliminary analysis. Statistical heterogeneity was evaluated using I-squared (I^2^) and chi-square statistics. High I^2^ values suggested considerable between-study variability, warranting the use of a random-effects model. Leave-one-out sensitivity analysis was conducted to assess the impact of individual studies on the overall meta-analysis.

## Results

### Search results and baseline characteristics of included studies

**Figure 1:**
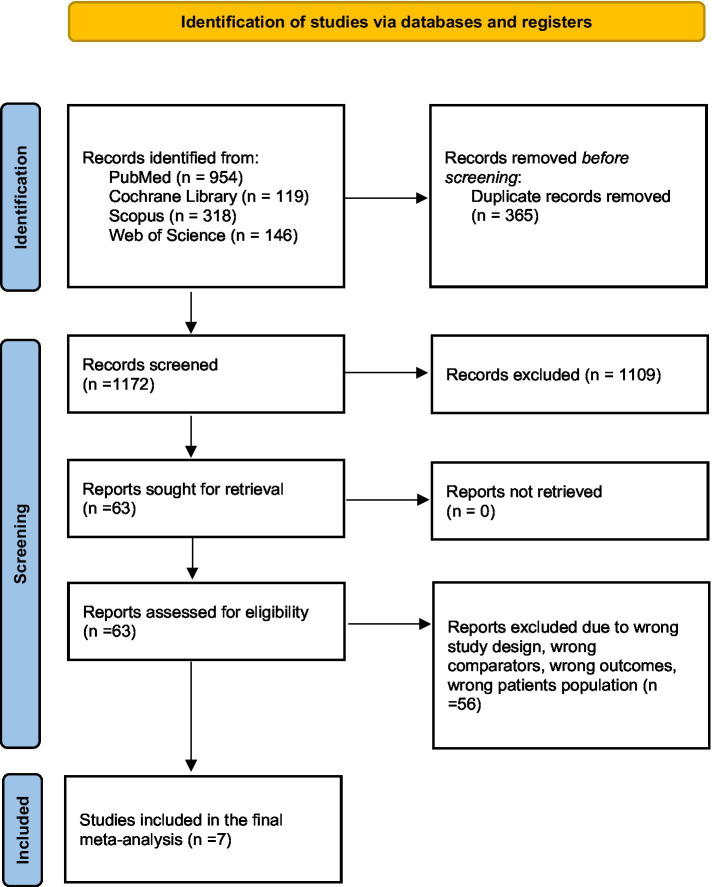
PRISMA flow diagram.

We retrieved 1,573 articles from database records. In total, 365 duplicates were detected. Title and abstract screening was performed for 1,172 records, and only 63 studies were eligible for full-text screening. Seven studies (Hamm et al., 2019; Kim et al., 2020; Zhen et al., 2020; Gao et al., 2021; Nishida et al., 2022; Liu et al., 2023; Urhuț et al., 2023) were included in the meta-analysis, as shown in the PRISMA flow diagram (Figure 1) (Page et al., 2020).

### General characteristics of the included studies

The general characteristics of the included studies are shown in Table 1. Seven studies demonstrated the potential of AI tools for improving the accuracy and efficiency of HCC diagnosis. Liu et al. (2023) established a Faster Region-based Convolutional Neural Network (RCNN) model for the differential diagnosis of primary clear cell carcinoma of the liver and common hepatocellular carcinoma (CHCC) (Liu et al., 2023). Similarly, Gao et al. (2021) developed an automatic diagnostic model to differentiate the types of malignant hepatic tumors based on multiphase contrast-enhanced computed tomography (CECT) and clinical data (Gao et al., 2021). Hamm et al. (2019) developed a custom convolutional neural network (CNN) model for classifying hepatic lesions on multiphasic MR images (Hamm et al., 2019). Kim et al. (2020) utilized a fine-tuned CNN to develop a deep learning model for detecting HCC using contrast-enhanced magnetic resonance imaging (MRI) (Kim et al., 2020). Urhuț et al. (2023) evaluated the accuracy of an automated method for classifying liver lesions using contrast-enhanced ultrasound (CEUS) (Urhuț et al., 2023). Nishida et al. (2022) constructed AI models for diagnosing liver tumors using B-mode ultrasonography, specifically CNNs, based on the visual geometry group network (VGGNet) model (Nishida et al., 2022). Finally, Zhen et al. (2020) developed a deep learning system (DLS) for classifying liver tumors based on enhanced and unenhanced MR and clinical data using CNNs based on the Inception-ResNet V2 network (Zhen et al., 2020).

**Table 1:** General characteristics of the included studies.

Ref.	Country	Sample size (total)	Title	Aim of the study	Diagnostic techniques used	AI tool used	Limitations of the study
Liu et al. (2023)	China	30 patients	Diagnosis of primary clear cell carcinoma of the liver based on Faster RCNN	Establish a Faster RCNN model for differential diagnosis of PCCCL and CHCC	Deep learning analysis of CT images	Faster RCNN	Single center study. -The sample size of the patients with PCCCL was small.
Gao et al. (2021)	China	159 patients	Deep learning for differential diagnosis of malignant hepatic tumors based on multiphase CECT and clinical data	Develop an automatic diagnostic model to differentiate types of malignant hepatic tumors	Multiphase CECT	Deep learning model (STIC)	Single-center study. -A limited number of imaging studies. Only typical lesions on MRI were used, excluding lesions with poor quality and more complex lesion types such as infiltrative HCC or complicated cysts. -Pathological proof was not available for all lesions.
Hamm et al. (2019)	USA	296 patients	Deep learning for liver tumor diagnosis part I: development of a CNN classifier for multiphasic MRI	Develop a CNN for classifying hepatic lesions on multiphasic MRI	Multiphasic MRI	Custom CNN	Relatively small training and testing dataset. -The included group was heterogeneous in terms of tumor types. -Insufficient number for some categories, such as focal nodular hyperplasia, liver abscess, liver adenoma, and cholangiocarcinoma. -Using only one type of ultrasound equipment and a single contrast agent for ultrasound. -Valuable information collected in daily clinical practice, such as tumoral markers, was not integrated.
Kim et al. (2020)	South Korea	950 images	Detection of Hepatocellular Carcinoma in Contrast-Enhanced MRI Using Deep Learning Classifier	Develop a deep learning model for detecting HCC using MRI	Contrast-enhanced MRI	Fine-tuned CNN	The image quality of the arterial phase was affected by transient severe motion artifacts. -The training dataset was obtained from a single vendor. -The study population had relatively good liver function. -The model is unable to detect atypical HCCs and low signal intensity in hepatobiliary phase MRI.
Urhuț et al. (2023)	Romania	49 patients	Diagnostic Performance of an AI Model Based on CEUS in Patients with Liver Lesions	Evaluate the accuracy of an automated method for classifying liver lesions using CEUS	CEUS (contrast-enhance ultrasound)	AI system based on algorithms	Single center study. -Lesions segmentation in the training validation set was done manually by doctors.
Nishida et al. (2022)	Japan	55 patients	Artificial intelligence models for the ultrasonographic diagnosis of liver tumors	Construct AI models for diagnosing liver tumors using ultrasonography	B-mode ultrasonography	CNNs based on VGGNet	A single-center retrospective study. -Patients who have specific types of focal liver diseases (small HCC, HCC without pathology, inflammation, etc.) need to be included in future training.
Zhen et al. (2020)	China	201 patients	Deep Learning for Accurate Diagnosis of Liver Tumor Based on MRI and Clinical Data	Develop a DLS for classifying liver tumors based on MRI and clinical data	Enhanced and unenhanced MRI	CNNs based on Inception-ResNet V2	The AI model focuses on diagnosis not detection. -Other types of rare liver tumors were not involved in the training set.

### Quality assessment

**Figure 2:**
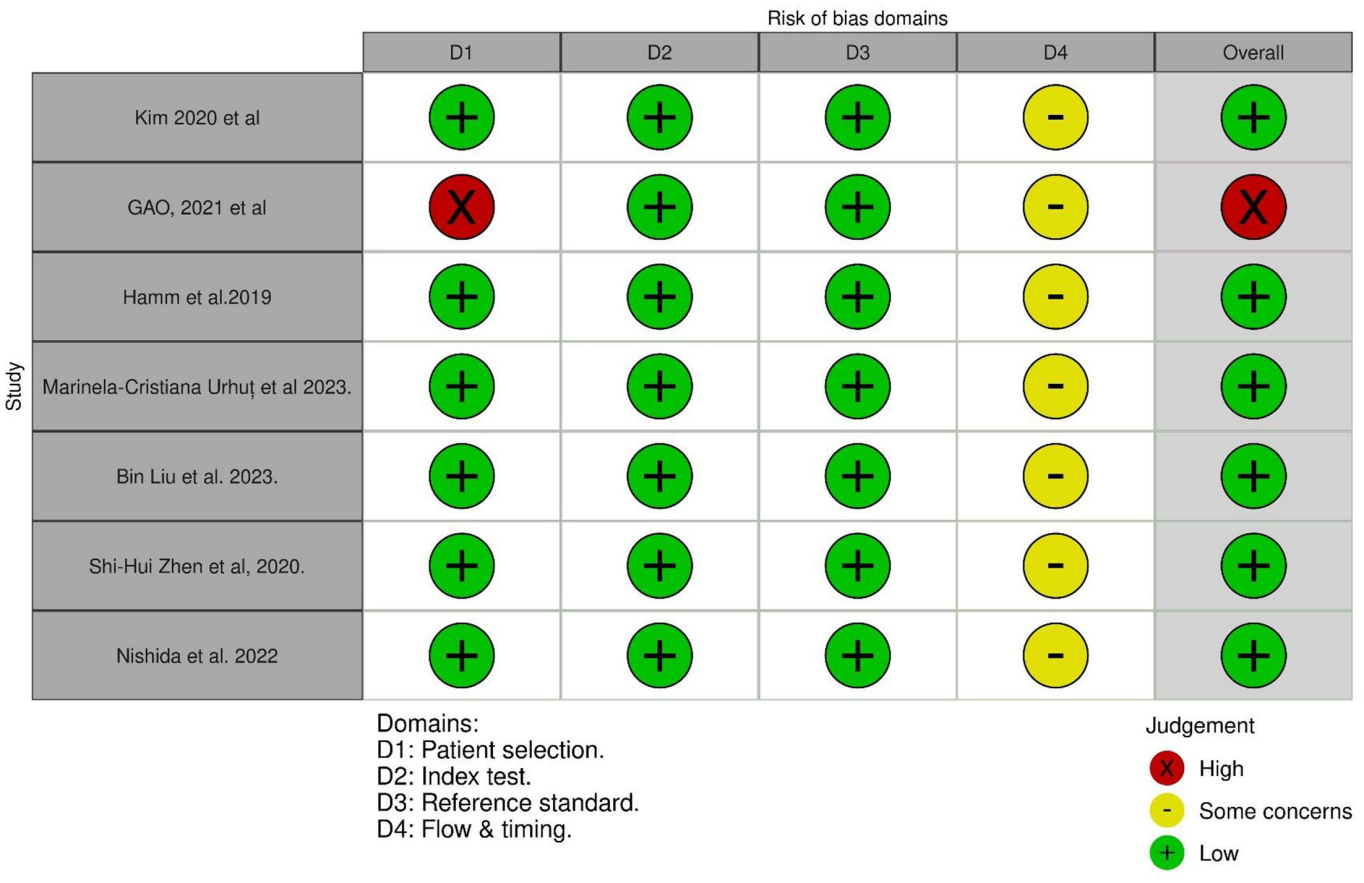
Quality assessment of the included studies.

All studies had a low risk of bias in patient selection, index tests, and reference standards, except for Gao et al. (2021). Gao et al. (2021) reported a high risk of bias in the patient selection domain. All studies had concerns regarding the flow and timing domains, as the reported process was not sufficient to judge this domain. Overall, Gao et al. (2021) had a high risk of bias, while the other studies had a low risk (Figure 2).

## Results

First of all, it is necessary to outline and explain the parameters of common effect and random effects models employed in the analysis. The fixed-effect model, also known as common-effect model assumes one true effect size underlying all studies included in this meta-analysis. In cases where studies are assumed to be similar enough in design and population, that’s when this model is appropriate. The key parameter for this particular model is pooled effect size which represents average of the sizes of the effects obtained from all the studies.

Contrarily, random-effects model believes that there is a difference in true effect sizes among studies. It takes into consideration variability within and between variables across various research thus becoming an appropriate choice if considerable heterogeneity exists amongst several researches. Within this specific model the vital parameters include pooled effect size and between-study variance commonly referred to as Tau^2^ indicating how much there exists variation amidst true effects.

The between-study variance in a meta-analysis is calculated by Tau^2^ (Tau-squared) and it helps us understand how much the effect sizes vary across studies over and above what would be expected by chance alone. Although there is no exact cutoff, we can interpret small heterogeneity of values near 0, moderate heterogeneity ranging from 0.01 to 0.1, and large heterogeneity indicated by values greater than 0.1 (West et al., 2010). For example, in this context, a Tau^2^ value of 0.0057 indicates that there is moderate heterogeneity which implies that while there may be some variation of the effect size found across the studies; it is not excessive enough to be called very high heterogeneity as such. In other words, differences between results of these studies are due to more than random occurrences but are not too far apart.

### Sensitivity

**Figure 3:**
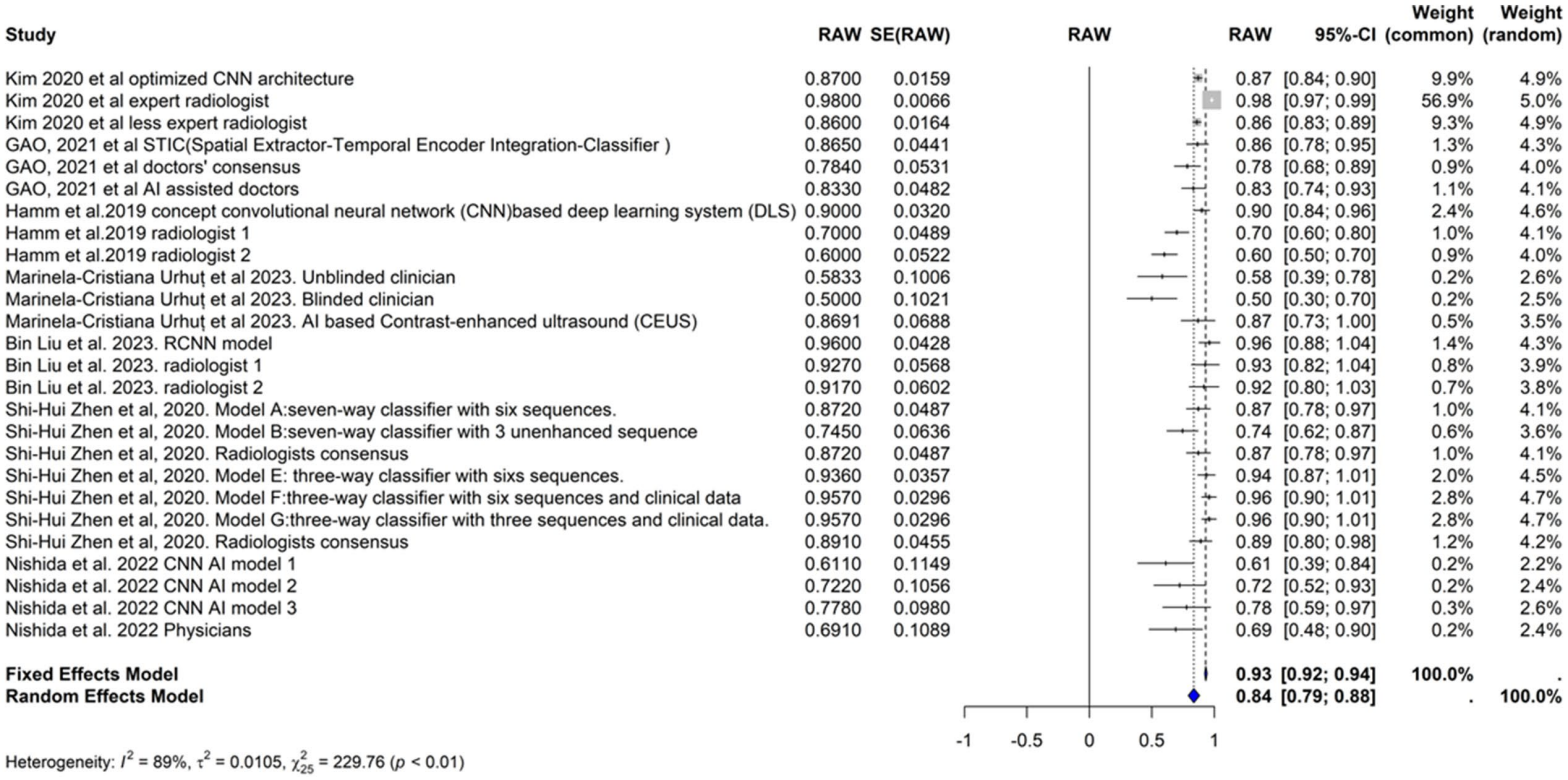
Forst plot of sensitivity analysis for AI models in hepatocellular carcinoma diagnosis.

We included the sensitivities reported in seven studies. Figure 3 shows the meta-analysis of 26 arm. We tested the included studies in the random and fixed effects models. According to the fixed effect model, the pooled sensitivity was 0.9317 (95% CI [0.9219, 0.9415]), suggesting high consistency across studies assuming a single underlying effect. The sensitivity of the random effects model was 0.8360 (95% CI [0.7909, 0.8811]), which accounts for the observed heterogeneity among studies and indicates a broader range of effect sizes. The average sensitivity of the AI-based models or physicians was 0.93, highlighting that the AI models, on average, perform similarly to physicians under the fixed effects model assumption, but these results showed more variability under the random effects model.

The performance of the AI models varied significantly depending on the specific model and diagnostic technique used. Some models, such as the RCNN model and the various classifiers used in Zhen et al. (2020), exhibited particularly high sensitivities. In contrast, the CNN-based models reported by Nishida et al. (2022) demonstrated lower sensitivities.

**Figure 4:**
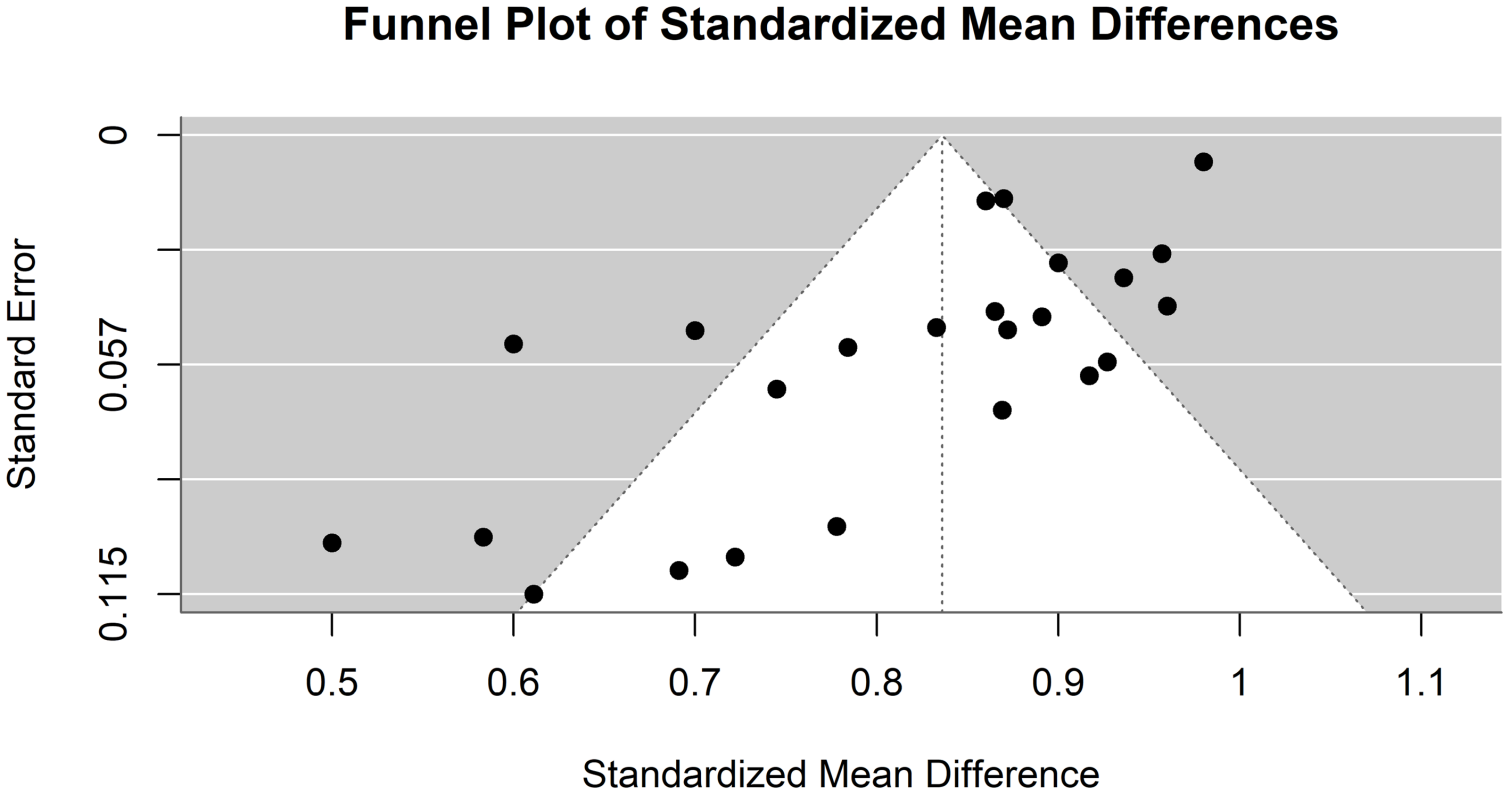
Funnel plot demonstrating publication bias in the meta-analysis of sensitivity across various models for hepatocellular carcinoma detection.

The heterogeneity of these studies was high. The I^2^ statistic was 89.1%, indicating a large variation among the studies’ estimates not only due to random error. The heterogeneity test was highly significant (Q = 229.76, df. = 25, *p* value <0.0001). The Tau^2^ statistic was 0.0105 (95% CI [0.0058–0.0277]), supporting moderate heterogeneity and reinforcing the need for a random-effects model to accurately capture the variability in sensitivities among different studies. The leave-one-out sensitivity analysis revealed that the pooled sensitivity estimates of the fixed effects model ranged from 0.8678 to 0.9390, and those of the random effects model ranged from 0.8292 to 0.8506, which confirmed the robustness of our findings, with pooled sensitivity estimates remaining stable across different models. These results suggest that no single study unduly influenced the meta-analysis results. Figure 4 represents the funnel plot of the included studies, suggesting potential publication bias.

### Specificity

**Figure 5:**
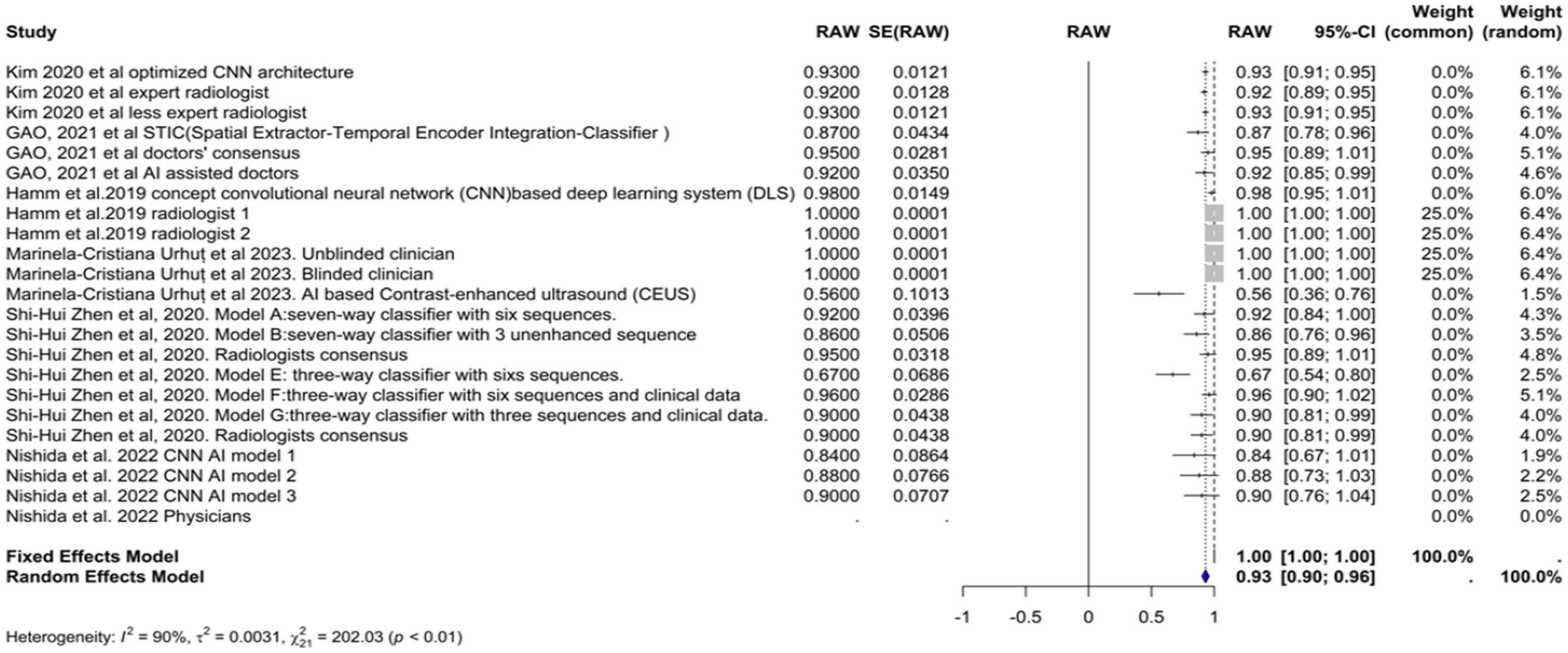
Forst plot of specificity analysis for AI models in hepatocellular carcinoma diagnosis.

**Figure 6:**
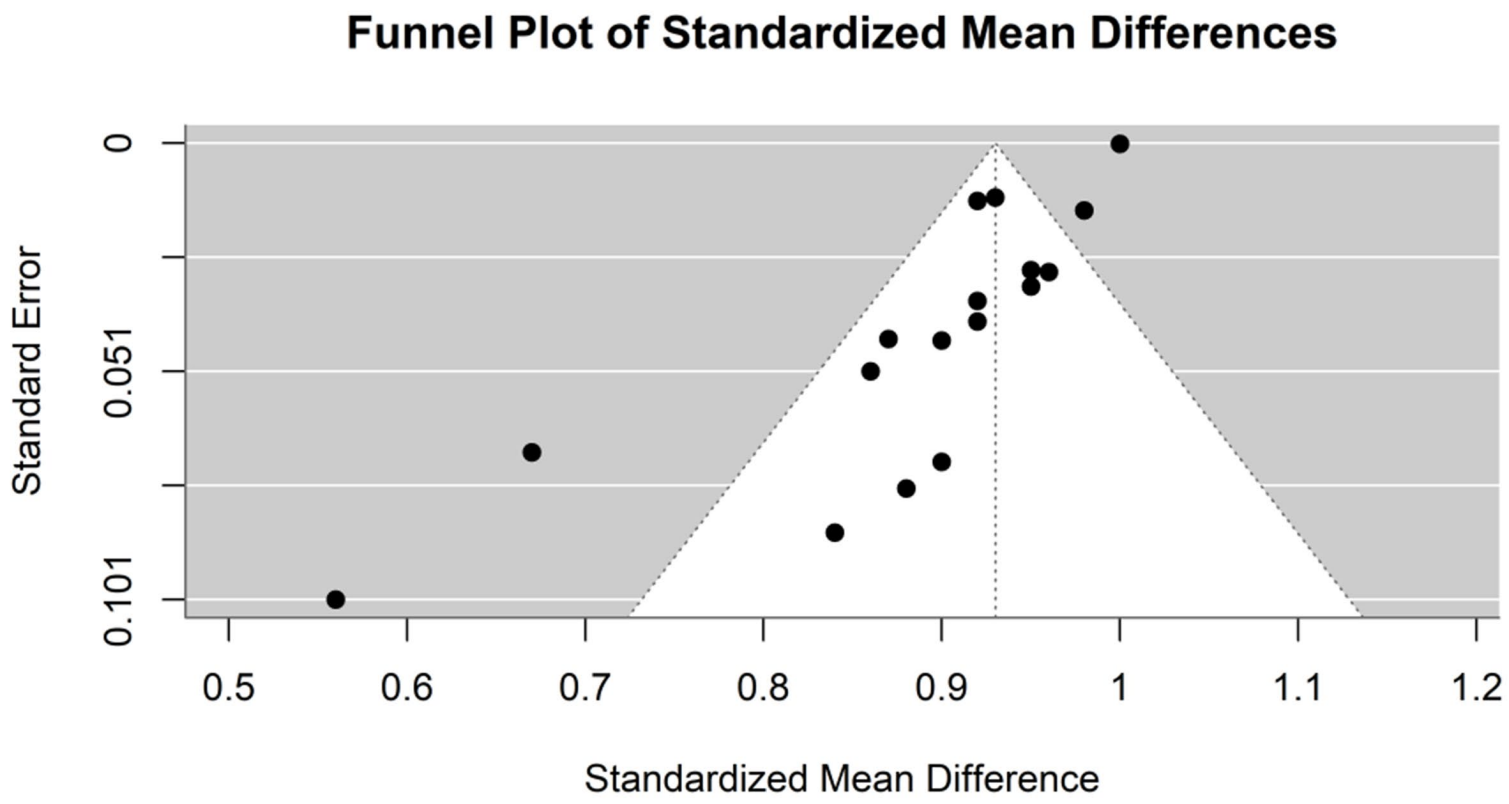
Funnel plot demonstrating publication bias in the meta-analysis of specificity across various models for hepatocellular carcinoma detection.

The specificity of the AI models used in the diagnosis of hepatocellular carcinoma (HCC) was evaluated in six studies (Figure 5). The common effect model showed a perfect specificity of 1.0000 (95% CI [0.9999, 1.0001]). The specificity in the random effects model was 0.9252 (95% CI [0.8915, 0.9589]), reflecting the variability among the studies. In Kim et al. (2020), the optimized CNN model had the same specificity as the expert radiologist (93%). Model F in Zhen et al. (2020) had the highest specificity (96.2%), while the model in Urhuț et al. (2023) had the lowest value (56.2%) (see Figure 6).

Heterogeneity among the studies was quantified using several statistical methods. The I^2^ statistic was 89.5%, which indicates that the total variation in the study estimates was due to heterogeneity rather than chance. The Tau^2^ statistic was 0.0035 (95% CI of [0.0024–0.0185]), which suggests moderate heterogeneity in specificity estimates across studies. This heterogeneity may be influenced by the varying odds of having HCC across these studies. Higher odds might reduce specificity due to the greater chance of false positives in a more homogenous patient population. The heterogeneity test was highly significant (Q = 209.54, df. = 22, *p* value <0.0001), confirming significant between-study variability, justifying the use of the random-effects model.

### Accuracy

**Figure 7:**
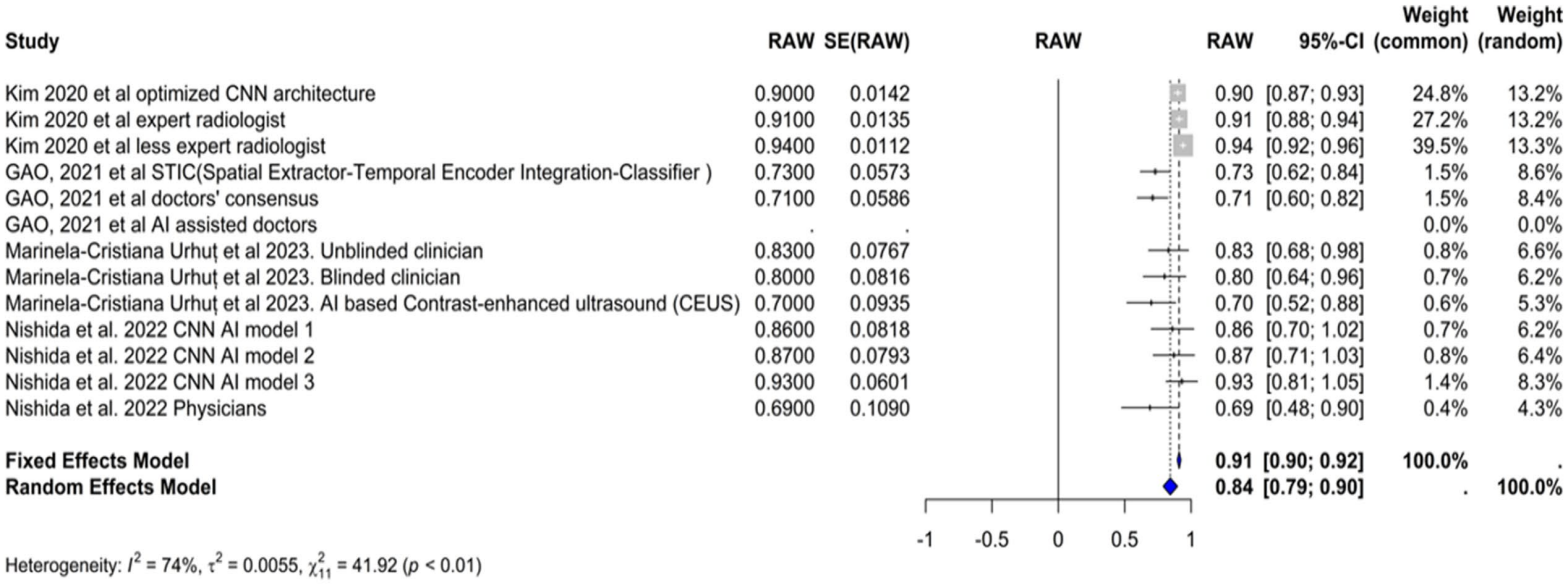
Forst plot of accuracy analysis for AI models in hepatocellular carcinoma diagnosis.

The accuracy of the AI models used in the diagnosis of hepatocellular carcinoma (HCC) was evaluated in four studies (Figure 7). The common effect model showed an accuracy of 0.9096 (95% CI [0.8958,0.9234]), suggesting high consistency across studies assuming a single underlying accuracy. The random effects model showed a slightly lower accuracy of 0.8423 (95% CI [0.7879–0.8966]). Most of the AI-based models achieved accuracies similar to or marginally better than those of physicians. Model 3 by Nishida et al. (2022) achieved the highest accuracy (92.7%), while the models of Kim et al. (2020) and Gao et al. (2021) had slightly better accuracy than did the other models (90% vs. 91 and 72.6% vs. 70.8%, respectively). The Urhuț et al. (2023) model had the lowest accuracy (69.6%), which was consistent with the specificity value. The I^2^ statistic was 74.1%, indicating that a large proportion of the total variation in the study estimates was due to heterogeneity rather than chance. The Tau^2^ statistic was 0.0057 (95% CI [0.0013, 0.0211]), highlighting moderate heterogeneity in the accuracy estimates across studies. This variability may be influenced by the differing odds of HCC among the study populations. Higher odds might lead to greater accuracy due to the increased incidence of the condition, while lower odds might result in lower accuracy as the model encounters more non-HCC patients. The heterogeneity test result was highly significant (Q = 42.53, df. = 11, *p* value <0.0001), supporting the use of the random-effects model due to significant between-study differences.

**Figure 8:**
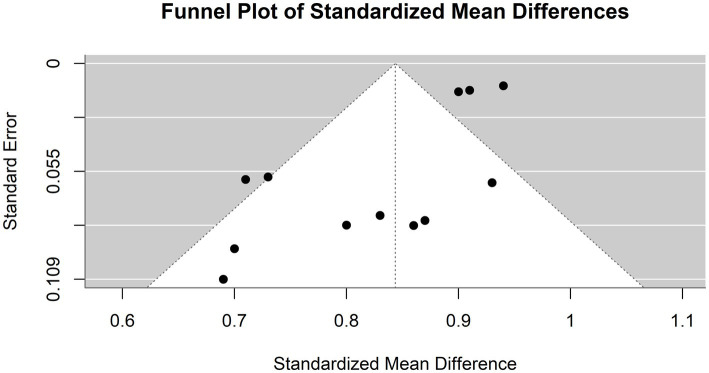
Funnel plot demonstrating publication bias in the meta-analysis of accuracy across various models for hepatocellular carcinoma detection.

Figure 8 illustrates a funnel plot utilized in the meta-analysis to examine potential publication bias; the funnel plot indicates potential publication bias among the included studies.

### Odds of HCC in study populations

To provide context for evaluating the models’ performance, the odds of having hepatocellular carcinoma (HCC) were calculated for each study population. In Liu et al. (2023), the odds of having HCC were infinite because the sample consisted entirely of HCC patients. According to Gao et al. (2021), the odds of having HCC are approximately 1.69. According to Hamm et al. (2019), the odds ratio was approximately 2.08. Kim et al. (2020) reported that the odds of having HCC were approximately 0.73. The odds ratio of Urhuț et al. (2023) was approximately 0.69. Nishida et al. (2022) reported odds of approximately 1.2%. Finally, Zhen et al. (2020) reported that the odds of having HCC were approximately 2.94 (Table 2).

**Table 2:** Odds of HCC in study populations.

Study	Arms	Odds ratio	Sample size	Sensitivity (95% CI)	Specificity (95% CI)	Accuracy (95% CI)
Kim et al. (2020)	Optimized CNN architecture	0.73	549	0.8700	0.93	0.90
	Expert radiologist			0.9800	0.92	0.91
	Less expert radiologist			0.8600	0.93	0.94
Gao et al. (2021)	STIC (Spatial Extractor-Temporal Encoder Integration-Classifier)	1.69	60	0.8650	0.87	0.73
	Doctors’ consensus			0.7840	0.95	0.71
	AI assisted doctors			0.8330	0.92	–
Hamm et al. (2019)	Concept convolutional neural network (CNN) based deep learning system (DLS)	2.08	88	0.9000	0.98	–
	Radiologist 1			0.7000	1.00	–
	Radiologist 2			0.6000	1.00	–
Urhuț et al. (2023)	Unblinded clinician	0.69	24	0.5833	1.00	0.83
	Blinded clinician			0.5000	1.00	0.80
	AI based Contrast-enhanced ultrasound (CEUS)			0.8691	0.56	0.70
Liu et al. (2023)	RCNN model	Infinite	21	0.9600	–	–
	Radiologist 1			0.9270	–	–
	Radiologist 2			0.9170	–	–
Zhen et al. (2020)	Model A: seven-way classifier with six sequences.	2.94	47	0.8720	0.92	–
	Model B: seven-way classifier with 3 unenhanced sequences			0.7450	0.86	–
	Radiologists’ consensus			0.8720	0.95	–
	Model E: three-way classifier with sixes sequences.			0.9360	0.67	–
	Model F: three-way classifier with six sequences and clinical data			0.9570	0.96	–
	Model G: three-way classifier with three sequences and clinical data.			0.9570	0.90	–
	Radiologists’ consensus			0.8910	0.90	–
Nishida et al. (2022)	CNN AI model 1	1.2	18	0.6110	0.84	0.86
	CNN AI model 2			0.7220	0.88	0.87
	CNN AI model 3			0.7780	0.90	0.93
	Physicians			0.6910	–	0.69

The varying odds of HCC across these studies could influence the reported sensitivities and specificities. For instance, Nishida et al. (2022) demonstrated lower sensitivities, which may be influenced by the odds of having HCC in their study population (approximately 1.2%). A greater risk of HCC might lead to greater sensitivity due to the increased incidence of this condition. In comparison, lower odds might result in lower sensitivity as the model encounters more non-HCC patients. Specificity may also be affected similarly, with higher odds potentially reducing specificity due to the greater chance of false positives in a more homogenous patient population.

## Discussion

In this systematic review and meta-analysis, we aimed to explore the potential of artificial intelligence tools for the diagnosis of hepatocellular carcinoma compared with human expertise. New models are being developed daily, which is considered an invaluable opportunity for advancing diagnostic accuracy and saving doctors’ time. Several scientists have debated whether these tools can replace humans in the future. Our study is the first systematic review to compare AI-based tools with physicians in the diagnosis of HCC. Early diagnosis of HCC is pivotal because patient survival is linked to hepatocellular carcinoma staging; patients diagnosed in early stages have higher five-year survival rates than those diagnosed in late stages (70 and 20%, respectively). Therefore, there is an urgent need to develop accurate and sensitive tools to optimize the diagnostic process for HCC (American Cancer Society, 2020).

Our analysis showed that most AI models are more sensitive than physicians, except for Kim et al. (2020), in which the expert radiologist had greater sensitivity than the AI model (98 vs. 87, respectively); however, they had almost the same specificity (93%). The proposed model had a sensitivity similar to that of a nonexpert radiologist (approximately 86.5%) (Kim et al., 2020). In some cases, the diagnosis of HCC using imaging techniques is quite challenging and requires strong experience, as there is great heterogeneity within HCC cells; different areas can have different growth patterns and levels of differentiation (Quaglia, 2018).

To address this issue, Zhen et al. (2020) developed an AI model that combines MR images and clinical data. This combination significantly improved the classification ability of the new models [AUC = 0.985 (95% CI, 0.960–1)]. Furthermore, Zhen et al. (2020) investigated the impact of sequence number on model performance. Different imaging sequences were used to train the model. Six sequences (contrast-enhanced T1 sequence, contrast-enhanced T1 sequence, diffusion, late arterial, portal venous, and equilibrium) or three sequences (T1, T2, and diffusion) as well as clinical data were utilized as model inputs. When clinical data were built into the model, the number of sequences did not have an impact on the performance. However, for models based on images only, model A, which had only three unenhanced sequences (AUC = 0.925, 95% CI = 0.871, 0.978), had better results than model B, which had 0.879, 95% CI = 0.813, 0.9452 (Zhen et al., 2020). Gao et al. (2021) measured the sensitivity and accuracy of AI-assisted physicians versus the AI model (STIC) or physicians alone. The AI-assisted physicians and the STIC model had approximately comparable sensitivities of 83.3 and 86.5%, respectively. Physicians had the lowest sensitivity (78.4%), but their accuracy did not significantly differ from that of the STIC model (70.8 and 72.6%, respectively) (Hamm et al., 2019; Kim et al., 2020; Zhen et al., 2020; Gao et al., 2021; Nishida et al., 2022; Liu et al., 2023; Urhuț et al., 2023).

The most sensitive model was the faster region-based convolutional neural network (RCNN) proposed by Liu et al. (2023). Models based on RCNNs exhibit a good ability to extract data from various images (Hamm et al., 2019; Kim et al., 2020; Zhen et al., 2020; Gao et al., 2021; Nishida et al., 2022; Liu et al., 2023; Urhuț et al., 2023). Raimundo et al. (2023) developed an AI model that combines RCCN and MRI for the diagnosis of breast cancer. Similarly, this model achieved a high accuracy of 94.4%. Another model by Liu et al. (2022) was developed based on the RCNN and medical images for diagnosing bile duct tumor thrombi in patients with HCC. In addition, this model showed a high sensitivity of 94% and a good specificity of 78%, which confirms the efficiency of RCNN-based models in improving the diagnosis of medical images (Liu et al., 2022, 2023; Raimundo et al., 2023).

Several diagnostic AI models based on convolutional neural networks (CNNs) have evolved because they do not require a clear definition of the lesion to interpret the images. Yamashita et al. (2020) proposed a CNN-based model that could classify hepatic observations without predefining hand-crafted imaging features with 60.4% accuracy. Similarly, Wang et al. (2019) investigated a convolutional neural network (CNN)-based model for the differentiation of liver masses via dynamic contrast agent-enhanced computed tomography (CT). The model achieved a median diagnostic accuracy of 0.84 (Yasaka et al., 2018).

Nishida et al. (2022) developed three different models to establish the relationship between the training dataset size and CNN performance. Model 3, which had the most training data, performed better than the other two models and the physicians did. Increasing the training set size has a proven impact on improving the accuracy of CNN-based models; nevertheless, large training sets consume more money, time, and effort (Radiuk, 2017; Nishida et al., 2022).

Clinical data have a major influence on imaging interpretation. Urhuț et al. (2023) compared the performance of novel AI-based contrast-enhanced ultrasound (CEUS) with that of two groups of clinicians: the first group knew all relevant clinical data, while the second group was blinded. The sensitivity and accuracy of the blinded group were lower than those of the unblinded group (58.3 and 83% vs. 50 and 79.6%, respectively). Furthermore, the AI-based contrast-enhanced ultrasound (CEUS) model had high sensitivity (86.9%) but less accuracy (69.9%) than did clinicians who were aware of the patient’s case (Urhuț et al., 2023). The sensitivity of contrast-enhanced ultrasound varies across different studies. A meta-analysis involving studies conducted in 1996–2016 showed that the sensitivity of CEUS for detecting HCC was 85%, although Jiang et al. (2021) reported a lower sensitivity of 69% for lesions <20 mm and 75% for lesions ≥20 mm. The combination of CEUS and AFP levels showed promising results, and the sensitivity increased to 83.1%; therefore, there is a need to establish evidence of the efficiency of CEUS in the diagnosis of HCC and to investigate the performance of the triple combination of CEUS, AFP levels, and AI models (Zhang et al., 2017; Jiang et al., 2021). In addition to diagnosing HCC, machine learning has a promising role in liver transplantation. Machine learning-based models could provide excellent predictions of the risk of complications and short-and long-term mortality. Furthermore, they can predict posttransplant outcomes better than traditional scoring systems. The incorporation of AI into the transplantation process saves time and money and increases the rate of transportation success (Chongo and Soldera, 2024). In general, AI has great potential in diagnosing different gastrointestinal tract pathologies due to its ability to use complex mathematical data involving multiple parameters and sophisticated formulas to draw conclusions that would be challenging or unfeasible for humans to process alone. However, many concerns are associated with the potential of AI in prognostication, such as the need for high-quality images for model training, ethical considerations of data usage, and the cost–benefit ratio (Do and Gastrointestinal, 2023).

### Limitations of the study

In our meta-analysis, high heterogeneity was observed, which may be attributed to the use of various AI models and diagnostic tools, in addition to differences in sample size. Some were single-center preliminary studies with small sample sizes, which is considered a major limitation. Another limitation is the inadequate inclusion of rare and complex liver lesions in the training and validation datasets of the AI models. There is a great need for randomized clinical trials with larger sample sizes. Moreover, developers should incorporate clinical history, laboratory findings, prior pathology reports, and immunohistochemistry tests to maximize model efficiency during the development of new AI models.

## Conclusion

Compared with physicians, AI models have the ability to improve the diagnosis of hepatocellular carcinoma. Faster R-CNN-based models are excellent for imaging classification and data extraction. CNN-based models have high sensitivity, and increasing the size of the training database significantly augments the accuracy of CNN models. The sensitivity of CEUS in the diagnosis of HCC is debatable; however, the combination of CEUS and AI models yields high sensitivity. Despite the promising results, AI models should not entirely replace humans in the diagnostic process; rather, they should be used as an assistant tool for more accurate and less timed decisions. We need to conduct more studies on the performance of AI-assisted physicians versus physicians without assistance, considering physicians’ level of experience.

## Author contributions

FA-O: Conceptualization, Data curation, Formal analysis, Funding acquisition, Investigation, Methodology, Project administration, Resources, Software, Supervision, Validation, Visualization, Writing – original draft, Writing – review & editing. WH: Conceptualization, Data curation, Formal analysis, Funding acquisition, Investigation, Methodology, Project administration, Resources, Software, Supervision, Validation, Visualization, Writing – original draft, Writing – review & editing. MG: Conceptualization, Data curation, Formal analysis, Funding acquisition, Investigation, Methodology, Project administration, Resources, Software, Supervision, Validation, Visualization, Writing – original draft, Writing – review & editing. NA: Conceptualization, Data curation, Formal analysis, Funding acquisition, Investigation, Methodology, Project administration, Resources, Software, Supervision, Validation, Visualization, Writing – original draft, Writing – review & editing. MA: Conceptualization, Data curation, Formal analysis, Funding acquisition, Investigation, Methodology, Project administration, Resources, Software, Supervision, Validation, Visualization, Writing – original draft, Writing – review & editing. AY: Data curation, Formal analysis, Validation, Software, Writing – review and editing. AR: Data curation, Formal analysis, Methodology, Validation, Software, Writing – review and editing.

## Glossary

HCC: Hepatocellular Carcinoma
AFP: Alpha-fetoprotein
DCP: Des-gamma-Carboxy Prothrombin
GP73: Golgi Protein 73
GPC3: Glypican-3
Ct-ncRNA: Circulating tumor noncoding RNA
CfDNA: Cell-free DNA
CtDNA: Circulating tumor DNA
CTC: Circulating tumor cell
FNA: Fine Needle Aspiration
WSI: Whole-Slide Imaging
CT: Computed Tomography
MRI: Magnetic Resonance Imaging
CNN: Convolutional Neural Network
AI: Artificial Intelligence
DL: Deep Learning
VGGNet: Visual Geometry Group Network
PRISMA: Preferred Reporting Items for Systematic Reviews and Meta-Analysis
RCNN: Region-based Convolutional Neural Network
CECT: Contrast-Enhanced Computed Tomography
PCCCL: Primary Clear Cell Carcinoma
CHCC: Common Hepatocellular Carcinoma
CEUS: Contrast-enhanced ultrasound
